# Amylin in the Periphery II: An Updated Mini-Review

**DOI:** 10.1100/tsw.2006.263

**Published:** 2006-12-15

**Authors:** Peter J. Wookey, Thomas A. Lutz, Sof Andrikopoulos

**Affiliations:** ^1^ Department of Medicine, University of Melbourne, Austin Health, Repatriation Campus, Heidelberg Heights, Vic., Australia; ^2^ Institute of Veterinary Physiology Zurich Center for Integrative Human Physiology, University of Zurich, Switzerland

**Keywords:** amylin, hormone, growth factor, amyloidogenesis, beta-cell integrity, diabetes, brain, feeding, kidney, receptors

## Abstract

Amylin is a polypeptide that is cosecreted with insulin from the β cells of the pancreas. Therefore, in states of diabetes in which the β-cell mass is largely depleted or dysfunctional, insulin and amylin secretion are also lost or dysregulated.While the soluble monomeric form of amylin acts as a hormone that alters physiological responses related to feeding and acts as a specific growth factor, there has been renewed interest in the less-soluble oligomeric and insoluble polymeric forms of human (also monkey and cat) amylin that may contribute to the establishment of a pathophysiological pathway to overt diabetes. With this discovery has grown the hope of minimizing, with appropriate therapy, these toxic forms to preserve the functional β-cell mass. Human β cells may also be more vulnerable to these forms and one risk factor, a higher fat diet, may promote toxic forms. The generation and utilities of transgenic rodent models, which express enhanced levels of human amylin, have been accompanied by strategies that may lead to the reduction of toxic forms and associated risk factors.The successful definition and faithful expression of the physiological receptors (and complexes) for amylin that may differ for each target organ is an important development in the field of amylin research generally. Besides the heuristic value for the understanding of the molecular biology of receptors, the opportunity to screen and identify nonpeptide analogues that bind the physiological receptors has important implications for biomedicine and clinical practice in relation to treatments for diabetic complications, bone diseases, and eating disorders. In particular, in their capacities to mimic the effects of amylin as a growth factor, amylin analogues may prove useful in the stimulation of β-cell mass (in conjunction with other factors), reduce the activity of the osteoclast population, and stimulate the regeneration of proximal tubules following toxic insult (and thus avoid the development of renal insufficiency).

## INTRODUCTION

Amylin, discovered in 1987[1,2], is a hormone cosecreted with insulin by the β cells of the pancreas and, therefore, has been considered a partner peptide in the etiology of diabetes-associated complications and related conditions. This mini-review is an update, based on a mini-review published 4 years ago[3], and focuses principally on current issues that challenge researchers in the field. There are several comprehensive reviews that include a more detailed assessment of our current knowledge of the hormonal effects and biochemistry of amylin[3,4,5,6,7,8]. It is true to add that the recent approval for the administration of amylin analogues to human diabetic subjects has sparked a good deal of fresh interest in the issues of "amylin in the periphery".

One of the key questions addressed is the role played by different forms of human islet amylin in the etiology of type II diabetes and the utility of transgenic rodent models to address that research question. A future outcome could be the selection of candidate compounds that reduce the deleterious effects or formation of the toxic form. Could these compounds be useful as preventative treatments for potential diabetic subjects to stave off overt diabetes?

In view of the potential role of monomeric amylin and other peptides to act in synergy as growth factors, particularly in the context of the maintenance of β-cell mass, bone density, and kidney function, can high-affinity, nonpeptide analogues be screened and cocktails assembled that could promote healthy tissues?

The effect of amylin on functions related to feeding (including gut motility), in particular satiation used in the sense of control of meal size, is mediated by the brain and is considered here in the context of the actions of several peptides that are components of the gut-brain axis. Scientists would like to understand the peptides' actions so that related drugs might be developed to ameliorate eating disorders, such as obesity.

The final issue presented in detail is the unresolved issue of the identity of amylin receptors. Clearly, our knowledge of the molecular components of amylin receptor(s) in each tissue will be important to deduce for other than heuristic reasons. It is likely that amylin receptors differ in the central nervous system (CNS), kidney, endocrine pancreas, and bone. The question here is whether these molecular entities can be reassembled in a form that is useful for the identification of nonpeptide agonists and antagonists for the amelioration of feeding disorders and complications associated with diabetes.

## AMYLOIDOGENESIS AND β-CELL RESPONSES

There are several biological issues that need to be considered before the strong correlation between amyloid formation and the etiology of diabetes (type II) can be understood. Some of these issues have been discussed in the previous mini-review[3].

First, in species that express forms of amylin prone to amyloidogenesis, the percent of diabetics (type II) with amyloid plaque found in the pancreas is very high, reaching 90–100% in humans, cats, and macaque monkeys, and islet amyloidosis in feline diabetes is associated with a mean loss of β-cell mass of 50%[9]. In this context, it has recently been proposed that an event prior to the process of islet amyloid formation seems to be more toxic to β-cells than the end result of the amyloid plaque (discussed below).

Furthermore β cells from different species seem to differ in their vulnerability to different insults including toxic amyloid entities[10]. Other factors that may lead to net β-cell death and consequent β-cell insufficiency include oxidative damage, changes in islet blood flow, cytokine toxicity, and certain dietary influences[11,12].

## TRANSGENIC RODENT MODELS THAT EXPRESS HUMAN AMYLIN

It is evident that not all species that express and secrete amylin form islet amyloid plaques. Early work demonstrated that humans, primates, and cats form islet amyloid associated with diabetes, while rats and mice were devoid of islet amyloid formation. This species difference was due to an amyloidogenic sequence in amylin necessary for the formation of amyloid plaques[13]. Since rodents did not form islet amyloid, a number of research groups generated transgenic mice that expressed the human amylin gene in islet β cells, but the initial studies on these mouse transgenic models (discussed in [3]) were disappointing, as no islet amyloid could be detected[14,15,16,17].

Various strategies were then considered to increase amylin secretion including breeding to homozygosity and the induction of insulin resistance with nicotinic acid or glucocorticoids, but islet amyloid was still not detected, indicating that hypersecretion of amylin alone is not sufficient for the formation of amyloid[10,18]. It was believed that other factors might be required for islet amyloid formation. In retrospect, this should not have been a surprise, since humans express and secrete amyloidogenic amylin that in most of us does not normally result in amyloid, unless diabetes intervenes.

It was by chance with the relocation of one of the human amylin transgenic mouse lines to another animal facility that resulted in the formation of histological islet amyloid plaques, similar to those observed in humans with type II diabetes[18]. This phenomenon appeared to be linked to an increase in dietary fat, which was subsequently confirmed in this[19] and another transgenic model[20].

Subsequent studies have shown that obesity induced by expression of the leptin mutation (*ob/ob*)[21], or ectopic and ubiquitous expression of agouti ($A^{vy}/a$)[22,23], resulted in islet amyloid formation associated with hyperglycemia. In fact, the latter study suggested that it was an event prior to islet amyloid formation and possibly intracellular, soluble amylin oligomers that were more toxic to islet β cells than the amyloid plaque[23]. The idea that oligomeric amylin is toxic is a recent development in the field that has been supported by research using a new model, the transgenic human amylin (HIP) rat[24,25]. This HIP rat is characterized by the development of diabetes due to islet β-cell apoptosis in the absence of islet amyloid, leading the authors to conclude that intracellular amylin oligomers may be responsible for the net death of β cells[24,26]. Further *in vitro* studies support the role of amylin oligomers as the primary cause of β-cell death via activation of the apoptotic pathway[27].

## OTHER FACTORS MAY ALSO MAKE MAJOR CONTRIBUTIONS TO β-CELL SURVIVAL AND/OR THE GENERATION OF AMYLIN-RELATED TOXICITY

The generation of toxic forms of amylin may be potentiated in the context of other pathophysiological events associated with pancreatic islets.

The renin-angiotensin system (RAS) has been shown to have several functions including inhibition of proliferation, induction of apoptosis, and generation of reactive oxygen species that may be deleterious to cell function[28]. Recent studies have shown that the components of the RAS (angiotensinogen, AT-1, and AT-2 receptors) are present in the endocrine pancreas[29,30]. We have shown that components of the RAS are up-regulated in pancreatic islets of the Zucker fat (ZDF) rat and are associated with intraislet fibrosis, apoptosis, and oxidative stress[31]. Pretreatment of ZDF rats with the angiotensin converting enzyme inhibitors perindopril or irbesartan decreased the components of the RAS, reduced islet fibrogenesis and apoptosis, and significantly improved glucose-mediated insulin secretion[31]. The impact of such regimens and altered islet blood flow on the generation of toxic forms of amylin are yet to be described.

## THE EFFECTS OF PERIPHERAL AMYLIN ON THE BRAIN AND THE GUT-BRAIN AXIS

The effects of peripheral amylin on the CNS have been described in terms of anorectic effects that alter physiological responses to feeding and putative dipsogenic effects that alter thirst and, therefore, drinking behavior[32]. The latter dipsogenic effects were exerted through neural pathways present in the subfornical organ (SFO) of the circumventricular organs (CVOs). Brain centers often activated in association with feeding, namely the lateral hypothalamic area (LHA) and the ventromedial hypothalamus (VMH), were also identified using functional MRI while drinking water[33].

In contrast to the limited description of the dipsogenic effects, there is now a large body of research on the effects of amylin in relation to feeding behavior including the well-documented promotion of satiation (that determines meal size), the inhibition of gastric emptying, and the control of glucagon secretion. Here the effects of amylin on satiation are introduced within the broader context of the gut-brain axis.

The original term “gut-brain” axis was coined in the late 1970s and resulted from discoveries that identified many of the gut peptides within the brain[34], previously considered quite separate compartments. Furthermore, the blood-brain barrier was considered an impenetrable barrier with regions of higher permeability typically only seen in certain anatomical locations such as the CVOs.

## EMERGING VIEWS OF THE “GUT-BRAIN” AXIS AND THE BLOOD-BRAIN BARRIER

More recently, the blood-brain barrier is emerging as a regulatory interface for many molecules including hormones and larger polypeptides[34]. In order to cross the blood-brain barrier, there are specific transporters (e.g., insulin, leptin), the activities of which may be modified and regulated by direct interactions with other serum proteins[35]. The activities of the specific transporters may be modified according to requirements to maintain the energy balance[36], the metabolic requirements of the CNS, activities within serum, and pathophysiological states, such as obesity and diabetes[37].

The full extent of the brain and neural networks that integrate feeding responses are part of a more general physiological concept of control of energy homeostasis, the neural components of which have yet to be defined fully. The systems that might be integrated at this level include conscious and conditioned responses that give rise to feeding behavior, control of energy reserves, circadian rhythms, sleep/wake cycles, and others.

An important site for the actions of amylin that alter feeding responses resides in the area postrema (AP) where calcitonin receptor (CTR)–positive neurons[38] are activated via a cGMP-dependent mechanism[39]. Surgical ablation of the AP resulted in the negation of the effects of salmon calcitonin (sCT) and amylin[40], and these actions have also been inhibited by the peptide antagonist of amylin or sCT binding, AC 187.

Amylin may act in synergy with other peptides of the gut-brain axis, such as cholecystokinin (CCK)[41,42,43], and such events may vary the amplitude of the response to amylin throughout the postprandial period.

Because of these effects of amylin that alter the feeding behavior, and in particular satiation that determines meal size, amylin analogues in combination with other components of the gut-brain axis are regarded as potential therapies for serious conditions related to obesity and possibly other eating disorders[6].

The brain centers that are activated following amylin actions at the AP have been described and include the nucleus of the solitary tract (NTS) and the lateral parabrachial nucleus (PBN), regions that have abundant CTR-positive neurons[38] (see also Figs. 1 and 2).

## AMYLIN AS A GROWTH FACTOR

Amylin mRNA transcripts (PJW, unpublished data) and protein[44,45] are expressed early in the latter half of gestation starting at embryo day 11/12 in the rat pancreatic diverticulum where amylin is expressed by P-cells[46]. These observations suggest that any function associated with the early fetal expression of this peptide may involve a role for amylin as a growth factor.

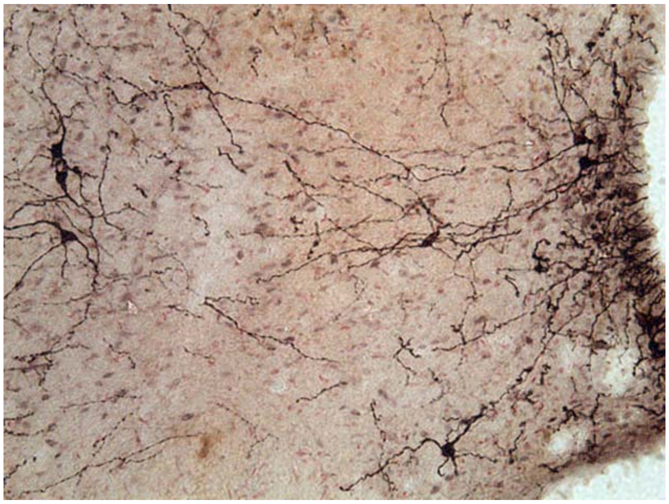

**FIGURE 1.** An example of CTR-positive neurons in the rat hindbrain that appear to form a network. Networks of calcitonin receptor (CTR)–positive neurons are often found[38] in key structures associated with energy homeostasis and feeding behavior including the NTS, paraventricular nucleus (PVN), lateral hypothalamic area (LHA), arcuate nucleus, as well as components of the limbic (nucleus accumbens), circadian (suprachiasmatic nucleus), and other systems (locus coeruleus). The relationship between CTR-positive neural networks and particular physiological functions has not yet been described.

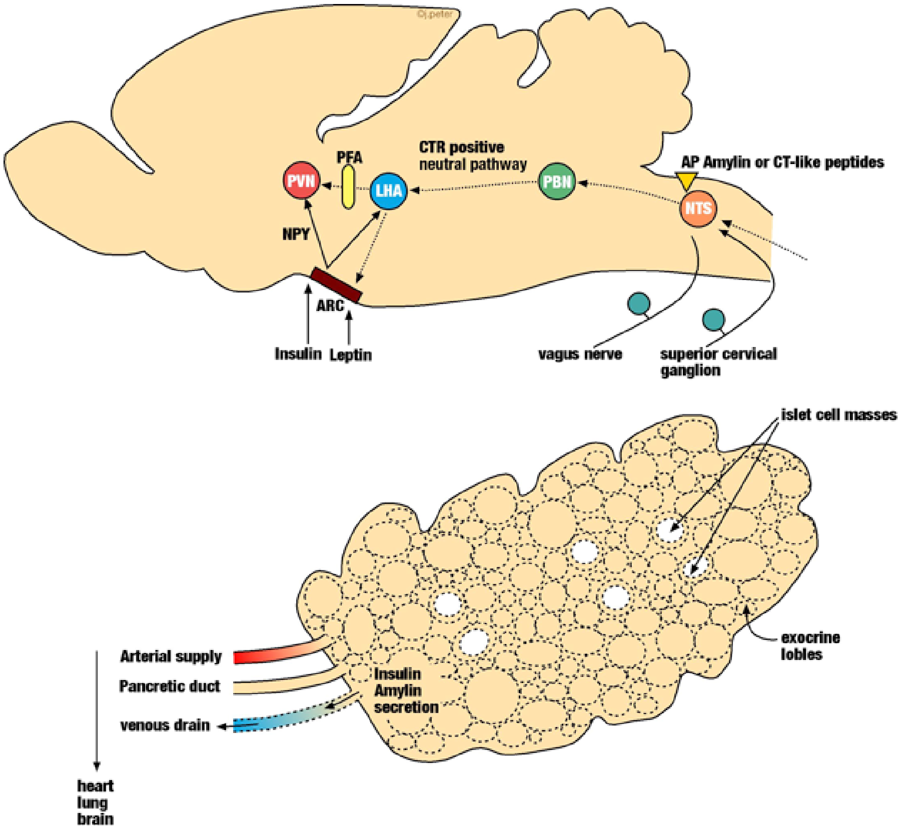

**FIGURE 2.** Cartoon of the neural and humoral relationship between the CNS and the endocrine pancreas. Abbreviations are area postrema (AP), nucleus of the solitary tract (NTS), parabrachial nucleus (PBN), lateral hypothalamic area (LHA), perifornical area (PFA), paraventricular nucleus (PVN), arcuate nucleus (ARC), neuropeptide Y (NPY).

## Pancreatic Islets

Monomeric amylin has been found to be a growth factor for isolated fetal β cells[48]. This follows previous descriptions of its effect on cells involved in bone metabolism and primary cultures of renal proximal tubular epithelial cells[49,50,51]. Furthermore, there is support for these data *in vivo*, in the observation that there is a large reduction in the β-cell mass measured in the AMY-/- diploid strain compared to wild-type controls[3].

These findings probably reflect the potential of monomeric amylin and it is likely that amylin acts in synergy with other growth factors such as somatostatin[52] by inducing proliferation of precursors of β cells and/or acting as a survival factor to improve the viability of adult β cells.

To understand the potential of monomeric amylin to act as a growth and/or survival factor, further research needs to be undertaken in the context of other events such as the role of cytokines such as IL-1[53], nitric oxide production[54], and the maintenance of islet blood flow.

## Renal Proximal Tubular Epithelial Cells

Amylin is also a potent proliferation factor both during the development of proximal tubules[55] and in the proliferation of adult epithelial cells[56]. The relevance of this action of amylin as a putative growth factor *in vivo* is further emphasized by the finding that amylin is expressed in the proximal tubules of the developing kidney. This occurs prior to vascularization of the rat kidney with peak levels of amylin mRNA at postnatal day 5 (PN5), some 200-fold higher than found in adult kidney[3]. This event is tightly regulated, a characteristic of the precise events that regulate growth factors during organogenesis.

The identification of the factors that control this tight regulation of expression of amylin may prove to be important later in pathogenic processes such as the development of renal complications associated with diabetes and renal regeneration following toxic or other insults.

## Osteoblasts and Osteoclast Differentiation

Research into the potential effects of amylin on bone density followed the observation that osteopenia was found in a large proportion of diabetic subjects[57,58,59,60]. Indeed, this potential effect of amylin was among one of the earliest effects described following its discovery. It was demonstrated subsequently that amylin acted as a growth factor in bone for the proliferation of osteoblasts[49,61] and recently in osteoclast differentiation[50].

More recently, the analyses of amylin and CTR gene-deletion mouse models[62] have demonstrated that amylin is a factor that inhibits osteoclastogenesis and, therefore, reduces the rate of osteolysis. In diabetics, the rate of bone loss is correspondingly accelerated.

On the other hand, as found in the CT gene-deletion model[63] and in the CTR+/- haplo-insufficient strain[62], there is an increase in the rate of bone formation that seems to contradict previous findings on the role of CT/CTR in the activity of osteoclasts.

## Dorsal Root Ganglia (DRG) in the AMY-/- Mouse Model

Amylin mRNA was detected in the developing DRGs of wild type fetal mice[64]. In addition, in the AMY-/- diploid strain the number of neuronal cell bodies in DRGs was depleted until around birth (PJW, unpublished observations). Together these observations suggested that any role of amylin as a growth factor for neurons in the DRGs was transitory or that it acted as a growth or tropic factor for a subgroup of neurons that were replaced in the DRGs around birth by other neuronal populations.

The latter may represent the truth since it was discovered that the adult AMY-/- diploid strain was deficient in aspects of nociception[65], suggesting permanent misrepresentation of a subgroup of neurons in the peripheral nervous system (PNS). Whether this is a mechanism that may help explain the changes in the PNS in diabetics has not yet been the subject of published research.

## THE MOLECULAR BIOLOGY OF GROUP B, G PROTEIN-COUPLED RECEPTORS (GPCRs)

Group B GPCRs include the seven transmembrane receptors for calcitonin (CTR), for adrenomedulin (AM) and calcitonin gene-related peptide (CGRP; calcitonin receptor-like receptor CRLR or CLR), vasoactive intestinal polypeptide/pituitary adenylyl cyclase activating peptide (VPAC1R), glucagon and parathyroid hormone (receptors PTHR1 and PTHR2), and several other peptide hormones[66].

In humans, there are several isoforms of CTR (hCTR), all of which result from gene-splicing events from a single CTR gene located on chromosome 7. The best characterized are the insert-minus (hCTRa) and insert-plus isoforms (hCTRb), which, in human CTR, includes a 16-amino residue insert towards the N-terminal domain of the putative second transmembrane span (span II) of hydrophobic amino residues. The isoform that may be regarded as equivalent in rodent (CTR C1b) includes a 31-amino residue sequence located after span II.

In either species, CTRa is the predominant isoform that is expressed by different subsets of cells within most tissues. Immunohistochemistry and anti-CTR antibodies have been successfully combined to identify these CTR-positive cells in a variety of physiological and pathophysiological states including cardiovascular disease and certain cancers (PJW, unpublished data).

Although the on and off rates for CT binding were similar, there are some clear differences in the characteristics of the two isoforms when expressed in the BHK cell line. First, hCTRb is significantly impaired in its rate of internalization[67]. Second, hCTRb displays a significantly reduced ability to couple to the second messenger enzymes adenylyl cyclase and phospholipase C. Third, stimulation of a transient calcium response was observed only with the hCTRa isoform[67].

Furthermore, when COS-7 cells were transfected with hCTRa, in contrast to the hCTRb isoform, the binding of the potent agonist salmon-CT resulted in retardation of the cell cycle with cells stalled in the G2/M phase[68]. A calcitonin response element (Sp1 binding site) was identified in the promoter of the human $p21^{WAF1/CIP1}$ gene encoding a cyclin-dependent kinase inhibitor[69].

## RECEPTOR ACTIVITY MODIFYING PROTEINS (RAMPs)

RAMPs interact with many members of the Group B GPCRs and appear to be generally involved in the cycling of these GPCRs to and from the plasma membrane destined for several cytoplasmic targets, depending on the type of RAMP (1, 2, or 3) heterodimer[70].

RAMP 1 combined with CLR results in a high-affinity CGRP receptor that can be inhibited with the nonpeptide antagonist BIBN4096BS, a drug developed for the inhibition of CGRP-mediated vasodilation, a common cause of migraine. RAMP 2 forms heterodimers with CLR to form the AM R1 receptor. RAMP 3 contains a PDZ motif near the C-terminus that is thought to direct the CLR-AM-RAMP3 complex to the lysozyme compartment for degradation. In this case, the receptor is degraded rather than being recycled to the plasma membrane[71].

The expression of RAMPs is far broader in tissues than the range of cell types that express CLR or CTR and, therefore, it was hypothesized that they may also dimerize with other GPCRs of Group B. In fact, it has been reported that RAMPs form heterodimers with VPAC1 receptor and the receptors PTHR1 and PTHR2[72]. In these instances, the association of RAMPs did not appear to influence binding, but rather internalization and recycling to the plasma membrane.

## THE ROLE OF GPCR RECEPTOR HOMO- AND HETERODIMERIZATION IN THE FORMATION OF HIGH-AFFINITY BINDING SITES

The classical idea that GPCRs function as monomeric entities has been unsettled by the emerging concept of GPCR homo- and heterodimerization[73]. Dimerization is a potential mechanism that could provide high-affinity binding sites for the list of CT-like peptides[74]. This list includes adrenomedulin-1 and -2, amylin, calcitonin, calcitonin gene-related peptides, and CTR-stimulating peptide[75].

Some of these hypothetical combinations of GPCR dimers may also provide lower-affinity binding sites that bind a broader spectrum of CT-like peptides.

## AMYLIN-LIKE RECEPTORS IDENTIFIED IN PHARMACOLOGICAL STUDIES USING CELL LINES

The interaction of RAMPs and CTR to form heterodimers that function as an amylin-like receptor *in vitro* is dependent on the isoform of CTR and the cell line used to express the components following transfection[66,76,77]. In COS-7 cells, the expression of hCTRb (in contrast to hCTRa) in conjunction with RAMPS resulted in the creation of an amylin-like receptor when [$^{125}$I]-rat amylin was used as the test ligand. On the other hand, in the cell line CHO-P, the coexpression of hCTRa and RAMP 2 resulted in the generation of an amylin-like binding site[66,78].

The interpretation of these data does not provide a clear idea of what events may contribute to create the putative amylin binding site, but does serve to raise several questions that are important to address before claims regarding the nature of the binding sites can be resolved.

First, as mentioned above there was a clear difference in the rates of internalization of hCTRa and hCTRb. The “binding” experiments[78] were carried out at 37ºC for 1 h on cells that were probably viable, but it is not clear from the data what proportion of the associated radioactive ligand was due to surface binding (exchangeable) or uptake. Second, the question arises: with coexpression of RAMPs, are there changes in the time-dependent rates of internalization of hCTRa and hCTRb that might influence uptake? Third, what effects do RAMPs have on the recruitment of G proteins, the coupling to second messenger enzymes, and the rate of phosphorylation/dephosphorylation that might influence the rate of recycling of these receptors, and hence the amount of associated radioactivity?

## THE PHYSIOLOGICAL ACTIONS OF AMYLIN *IN VIVO* AND EXPRESSION OF CTR

### Amylin Binding Sites in the Circumventricular Organs of the Brain

Within the brain, the AP has been identified at which amylin analogues interact to produce physiological responses related to feeding. Furthermore, the SFO has been identified as the region at which amylin and related peptides may exert dipsogenic actions[32,79].

The local expression in the CNS of cognate mRNAs for components thought to comprise an amylin receptor were identified, namely, the CTR C1b isoform and RAMP 2[80] and expression was investigated using the technique of *in situ* hybridization (ISH). It was also reported that c-fos mRNA was expressed in SFO neurons in response to the injection (*i.p.*) of amylin or sCT, supporting the SFO as a functional target for these peptides and implicating the expression of amylin receptors.

The same paper did not find significant evidence for expression of the isoform of CTR, C1b, in the AP of the hindbrain. This CVO has been identified as a crucial target for the activities of amylin in relation to feeding responses[40]. However, there is strong expression of CTR C1a in this region of the AP and NTS[48] that express increased levels of cGMP[39] following injection (*i.p.*) amylin or sCT, which is inhibited by the peptide analogue AC187.

## The Renal Sites of Amylin Actions

There are several studies that have focused on the actions of amylin *in vivo* and the possible involvement of CTRs, first in the kidney and second in bone metabolism.

In rat kidney, high-affinity, G protein-dependent binding sites for amylin were identified both using *in vitro*[81] and *in vivo* autoradiography[56], and these were localized to proximal tubules within the renal cortex. Furthermore, in micropuncture, split drop experiments amylin injected into the peritubular capillaries of proximal tubules resulted in stimulation of sodium reabsorption (28%) that was inhibited by amiloride, an inhibitor of the sodium/hydrogen exchanger[56]. Together, these studies identified proximal tubules of the renal cortex as the site of amylin actions in rat kidney.

In kidney, there are several independent studies that identify the sites of expression of CTR and [$^{125}$I]-salmon CT (sCT) binding sites. Salmon CT is a potent ligand of CTR and agonist of CTR actions that stimulates several second messenger systems and intracellular events (see above). CTR expression is found using immunohistochemistry, predominantly in the renal medulla[81] in epithelial cells of the distal tubules, loops of Henle, and collecting ducts, which is in agreement with earlier binding data that demonstrated binding to similar renal structures. The expression of RAMP 3 colocalized with CTR[3].

In summary, there is a clear distinction between the renal sites of amylin binding and actions on proximal tubules, and the sites of expression of CTR by epithelial cells of distal tubules, loops of Henle, and collecting ducts[82].

## Amylin Actions on Cells of Bone (In Vitro) and as Demonstrated in Mouse Genetic Models (In Vivo)

*In vitro* amylin has been characterized as a positive growth factor for osteoblasts[83] and a negative growth factor for osteoclasts[84]. This research provided the first evidence that amylin inhibits the terminal differentiation of osteoclasts essential for their activation of bone metabolism.

Thus, in mice that are wild type (AMY +/+), the rate of terminal differentiation of osteoclasts (involving fusion of mononuclear precursors into mature multinucleated osteoclasts) is reduced and bone mass is relatively high. In both AMY -/- and the haploid AMY +/- gene deletion mouse models in which amylin levels are reduced, bone mass is reduced due to the enhanced numbers and the total activity of terminally differentiated osteoclasts. The pathology of bones in this model is similar to the phenotype observed in osteoporosis. These actions may also explain the high prevalence of low bone mass among type I diabetic subjects[85].

In this study, it was noted that in the CTR-/- gene deletion model fetuses died early *in utero* prior to skeletogenesis, presumably because of the important role of CTR and endogenous ligands in development of fetal organs[86,87], for instance in CNS[88] and other fetal tissues (PJW, unpublished data) including perinatal bone.

Of importance here, it was noted that in the haploid CTR+/- bone density was greater than in wild type and that the bone formation rate (BFR) was 30% higher. The opposite effects on bone histology of haploinsufficiency in the AMY+/- and CTR+/- strain represents compelling evidence that amylin does not exert its effects via CTR in this case.

The separation of pathways of action was confirmed in the double AMY+/-, CTR+/- strain since there was both an increase in osteoclast numbers and an increase in the BFR.

One striking event that has yet to be understood in terms of the humoral factors that are involved is the acute postprandial decrease in bone resorption[89]. In view of the findings discussed above, one candidate might be amylin, perhaps acting in synergy with other hormones that are released in response to nutrient intake[89].

## THE IMPORTANCE OF AMYLIN RECEPTORS

Although it has been claimed that the proposed CTR/RAMP heterodimer solves the enigma of the amylin receptor[90], the topic is still far from resolved within the context of the physiological roles of amylin established so far, and the pharmacological data are enshrouded by the uncertainty of clear interpretation.

An important step forward in the quest to understand the actions of amylin, and for the translation of research on the molecular biology and pharmacology of amylin and its receptors will undoubtedly be the characterization of several nonpeptide agonists and antagonists as candidates for human therapies and further research on amylin receptors.

Given the important roles ascribed for amylin and its analogues in the control of the postprandial glucose surge in diabetic subjects, satiation, bone formation, and renal development and functions, the issue of the molecular identities of amylin receptors (and complexes) is compelling. The final solution may well reside in the definition of the active heterodimers of GPCRs that may also vary according to the particular tissue (brain, islets, bone, kidney, etc.).

## FUTURE DIRECTIONS OF BIOMEDICAL RESEARCH INTO AMYLIN-RELATED TOPICS

Amylin is expressed and largely conserved in molecular terms in each mammalian species, and without doubt plays significant roles related to physiological responses related to feeding and as a trophic factor. The latter role is relatively unexplored and may involve organ regeneration in adults following insult or provide a mechanism of cellular homeostasis in particular organs that are crucial for the maintenance of cellular functions, e.g., in the endocrine functions of the β-cell mass or renal proximal tubules.

The molecular composition and physiological outputs of amylin receptors will be important to refine, primarily for the selection of candidate nonpeptide agonists and antagonists that may aid in the treatment of existing diabetic conditions. These may improve postprandial control of glucose, help avoid hypoglycemic events, and might be coadministered with insulin. Furthermore, some candidates may have properties that reduce bone loss by restricting the numbers of functional osteoclasts particularly relevant to diabetic complications and osteopenia. They may also prove useful in the physiological integration of exogenous transplanted β cells to restore cellular sufficiency and viability. Finally, it is also possible that a cocktail of compounds including amylin analogues may prove vital in the treatment of obesity.

A further aspect requiring more refined pharmacological definition is the potentially toxic form of insoluble human amylin, its putative effects on apoptosis of β cells and maintenance of viable β-cell mass. Given the increasing incidence of diabetes (type II) there is a large incentive to discover compounds that ameliorate the pathophysiological development of these events that are currently regarded as central to the development of overt diabetes.

## ACKNOWLEDGMENTS

The help of Jeanne Peter in the preparation of Fig. 2 is gratefully acknowledged. Studies by TAL were sponsored by the Swiss National Science Foundation.

The original project undertaken by PJW was funded by the National Health and Medical Research Foundation, Australia.
